# Complete Genome Sequence of *Frog virus 3*, Isolated from a Strawberry Poison Frog (*Oophaga pumilio*) Imported from Nicaragua into the Netherlands

**DOI:** 10.1128/genomeA.00863-17

**Published:** 2017-08-31

**Authors:** Bernardo Saucedo, Joseph Hughes, Steven J. van Beurden, Nicolás M. Suárez, Olga L. M. Haenen, Michal Voorbergen-Laarman, Andrea Gröne, Marja J. L. Kik

**Affiliations:** a Utrecht University, Utrecht, the Netherlands; b MRC–University of Glasgow Centre for Virus Research, Glasgow, United Kingdom; c Wageningen Bioveterinary Research of Wageningen UR, Lelystad, the Netherlands

## Abstract

*Frog virus 3* was isolated from a strawberry poison frog (*Oophaga pumilio*) imported from Nicaragua via Germany to the Netherlands, and its complete genome sequence was determined. *Frog virus 3* isolate *Op*/2015/Netherlands/UU3150324001 is 107,183 bp long and has a nucleotide similarity of 98.26% to the reference *Frog virus 3* isolate.

## GENOME ANNOUNCEMENT

Ranaviruses (family *Iridoviridae*, subfamily *Alphairidovirinae*) have caused declines of poikilotherm populations worldwide (1–3). These viruses are commonly spread through international trade (4–8), sometimes resulting in the introduction of virulent strains into wild populations (9). In the Netherlands, there are two documented cases of ranaviruses in imported specimens; the first involved frog virus 3 (FV3) in red-tailed knobby newts (*Tylototriton kweichowensis*) from China (10), and the second involved common midwife toad virus (CMTV) in poison dart frogs (*Dendrobates auratus*, *Phyllobates bicolor*) from an undetermined location (11). Poison dart frogs imported from the Netherlands also experienced a frog virus 3-associated die-off upon arrival to Japan (12).

In 2015, two strawberry poison frogs (*Oophaga pumilio*), imported from Nicaragua via Germany, died upon arrival to the Netherlands. No gross lesions were observed, but histopathology revealed mild liver necrosis and intracytoplasmic basophilic inclusions in hepatocytes and bone marrow hematopoietic cells.

Conventional PCR and sequencing of the major capsid protein from liver samples of both animals revealed FV3, a clade of ranaviruses that, unlike CMTV, has not been reported to occur in Dutch nature (3, 13, 14). Subsequently, a 10% organ suspension from one animal was mixed with 1% antibiotics and inoculated on epithelioma papulosum cyprini cells. Once full cytopathic effect was observed, the virus was purified by high-speed ultracentrifugation on a 36% sucrose cushion as described previously (14). After resuspension in ice-cold phosphate-buffered saline (PBS), the DNA was extracted with the QIAamp DNA blood minikit (Qiagen) according to the manufacturer’s protocol. The DNA was sheared by sonication, and a library was prepared using the KAPA library preparation kit. A MiSeq system running a V3 chemistry platform (Illumina) was used to generate 2 × 300-nucleotide paired-end sequence reads. After quality control of the sequence reads using Trim Galore (http://github.com/FelixKrueger/TrimGalore), a *de novo* assembly using SPAdes (15) produced a contig of 107,183 bp with a total G+C content of 54.95%. Annotations were performed manually with ORF finder at the NCBI website to highlight putative protein products using the genome of the American FV3 isolate (FV3-reference; GenBank accession no. AY548484) as a reference (16).

The genomic structure of the FV3 *Oophaga pumilio* isolate (FV3-Op) showed a nucleotide similarity of 98.26% to the genome of FV3-reference and a 97.92% nucleotide similarity to FV3-SSME (KJ175144) (17). All 98 putative open reading frames present in other FV3-like virus counterparts were identified, with only a few features distinct from FV3-reference, including the lack of truncation in the eukaryotic initiation factor 2 alpha protein. It has been suggested that strains with the capacity to express the full version of this immunomodulatory protein are more pathogenic than those with a truncated version (18). Phylogenetic characterization using 45 ranavirus genes positioned FV3-Op in the FV3 clade, in a separate cluster from soft-shelled turtle iridovirus (EU627010.1) (19), *Rana grylio* iridovirus (JQ6545861.1) (20), and tiger frog virus (AF389451) (21). The isolation of FV3 from imported amphibians from Nicaragua highlights the constant risk of trade-associated introduction of foreign *Ranavirus* spp.

### Accession number(s).

This whole-genome shotgun project has been deposited in DDBJ/ENA/GenBank under the accession no. MF360246. The version described in this paper is the first version, MF360246.1.
